# Successive walnut plantations alter soil carbon quantity and quality by modifying microbial communities and enzyme activities

**DOI:** 10.3389/fmicb.2022.953552

**Published:** 2022-07-25

**Authors:** Haoan Luan, Yingru Liu, Shaohui Huang, Wenyan Qiao, Jie Chen, Tengfei Guo, Xiaojia Zhang, Suping Guo, Xuemei Zhang, Guohui Qi

**Affiliations:** ^1^College of Forestry, Hebei Agricultural University, Baoding, China; ^2^College of Agronomy, Hebei Agricultural University/North China Key Laboratory for Crop Germplasm Resources, Ministry of Education/State Key Laboratory of North China Crop Improvement and Regulation, Baoding, China; ^3^Hebei Fertilizer Technology Innovation Centre, Institute of Agricultural Resources and Environment, Hebei Academy of Agriculture and Forestry Sciences, Shijiazhuang, China; ^4^Institute of Agricultural Resources and Regional Planning/Key Laboratory of Plant Nutrition and Fertilizer of Ministry of Agriculture and Rural Affairs, Chinese Academy of Agricultural Sciences, Beijing, China; ^5^Institution of Plant Nutrition and Environmental Resources, Henan Academy of Agricultural Sciences, Zhengzhou, China; ^6^Tea Research Institute, Shandong Academy of Agricultural Sciences, Jinan, China

**Keywords:** walnut plantation ages, soil depths, soil microbial community, extracellular enzyme activities, soil organic carbon quantity, quality

## Abstract

Knowledge of the spatial–temporal variations of soil organic carbon (SOC) quantity and quality and its microbial regulation mechanisms is essential for long-term SOC sequestration in agroecosystems; nevertheless, this information is lacking in the process of walnut plantations. Here, we used the modified Walkley-Black method, phospholipid fatty acid analysis, and micro-plate enzyme technique to analyze the evolution of SOC stocks and quality/lability as well as microbial communities and enzyme activities at different soil depths in walnut plantations with a chronosequence of 0-, 7-, 14-, and 21-years in the Eastern Taihang Mountains, China. The results indicated that long-term walnut plantations (14-and 21-years) enhanced SOC stocks, improved SOC quality/lability (as indicated by the lability index), and promoted microbial growth and activities (i.e., hydrolase and oxidase activities) in the 0–40 cm soil layers. Besides, these above-mentioned SOC-and microbial-related indices (except for oxidase activities) decreased with increasing soil depths, while oxidase activities were higher in deeper soils (40–60 cm) than in other soils (0–40 cm). The partial least squares path model also revealed that walnut plantation ages and soil depths had positive and negative effects on microbial attributes (e.g., enzyme activities, fungal and bacterial communities), respectively. Meanwhile, the SOC stocks were closely related to the fungal community; meanwhile, the bacterial community affected SOC quality/liability by regulating enzyme activities. Comprehensively, long-term walnut plantations were conducive to increasing SOC stocks and quality through altering microbial communities and activities in the East Taihang Mountains in Hebei, China.

## Introduction

Soil organic carbon (SOC) is regarded as the most complex and less understood component of soil, which plays an essential role in sustaining soil fertility and agricultural production (Thomson et al., 2013; Liu et al., 2018). Also, Amelung et al. (2020) revealed that augmenting SOC stocks is beneficial for improving soil quality and mitigating climate warming. Yet, it is hard to disentangle the gains and losses of SOC in a short time because SOC has a low natural variability, along with a great background storage (Chaudhary et al., 2017). Moreover, Lehmann and Kleber (2015) indicates that SOC is a continuum of progressively decomposing organic constitutes and composed of various fractions with different properties. Therefore, to deeply understand SOC dynamics in agricultural soils, it is necessary to separate SOC into various C fractions with distinct characteristics (Paul, 2016).

In general, SOC can be divided into two kinds of pools, including labile/active C and stable/passive C (Zhang et al., 2022). Labile/active C pools, characterized by shorter (annual to decadal time scales) turnover times in soils, are vulnerable to agricultural management practices and can be regarded as indicators for assessing future SOC changes (Duval et al., 2018). Conversely, stable/passive C pools are hardly degraded and usually have longer turnover times, thus being conducive to long-term C storage (Purakayastha et al., 2008). Clarifying their quantity and proportions can provide more information about SOC characteristics (e.g., SOC quality and lability, etc.; Liu et al., 2018). Studies have previously reported that the regulatory factors for SOC characteristics (e.g., SOC quantity and quality), such as microbial attributes (Jiang et al., 2018), edaphic and environmental conditions (e.g., soil pH, moisture, and temperature; Cates et al., 2022), are intricate and variable. Among the above-mentioned variables, due to the critical roles of microbial contribution in the balance between SOC formation and degradation in soils (Trivedi et al., 2013), a deeper mechanistic understanding of microbial-driven changes in SOC quantity and quality is necessary.

In agroecosystems, a series of critical ecological processes (e.g., SOC dynamics and nutrient cycling) are performed by soil microbes (Jiang et al., 2018; Tian et al., 2021); meanwhile, the healthy microbial function was beneficial for several ecosystem services, including soil fertility and quality, etc. (Liu et al., 2022). Soil extracellular enzymes (EEs), which were primarily produced by microbes, could offer a ‘functional fingerprint’ for microbes due to their catabolic capabilities (Burns et al., 2013; Luo et al., 2017). Besides, several studies indicated that two functional microbial subgroups, *viz.*, fungi that utilize stable C resources, and bacteria that quickly metabolize labile C resources, are responsible for SOC formation and decomposition (Burns et al., 2013; Bei et al., 2022). Notably, most studies have reported the importance of microbes on SOC cycling through their physiological characteristics and metabolic functions (Alves et al., 2021; Zheng et al., 2022), with little attention given to microbial-driven variations in SOC quality. This less knowledge has appeared as one of the important uncertainties in our understanding of agricultural SOC sequestration to some extent. It is therefore imperative that we better understand the microbial significance in agricultural SOC dynamics through the combined study of microbes, EEs, and SOC pools.

**Figure 1:**
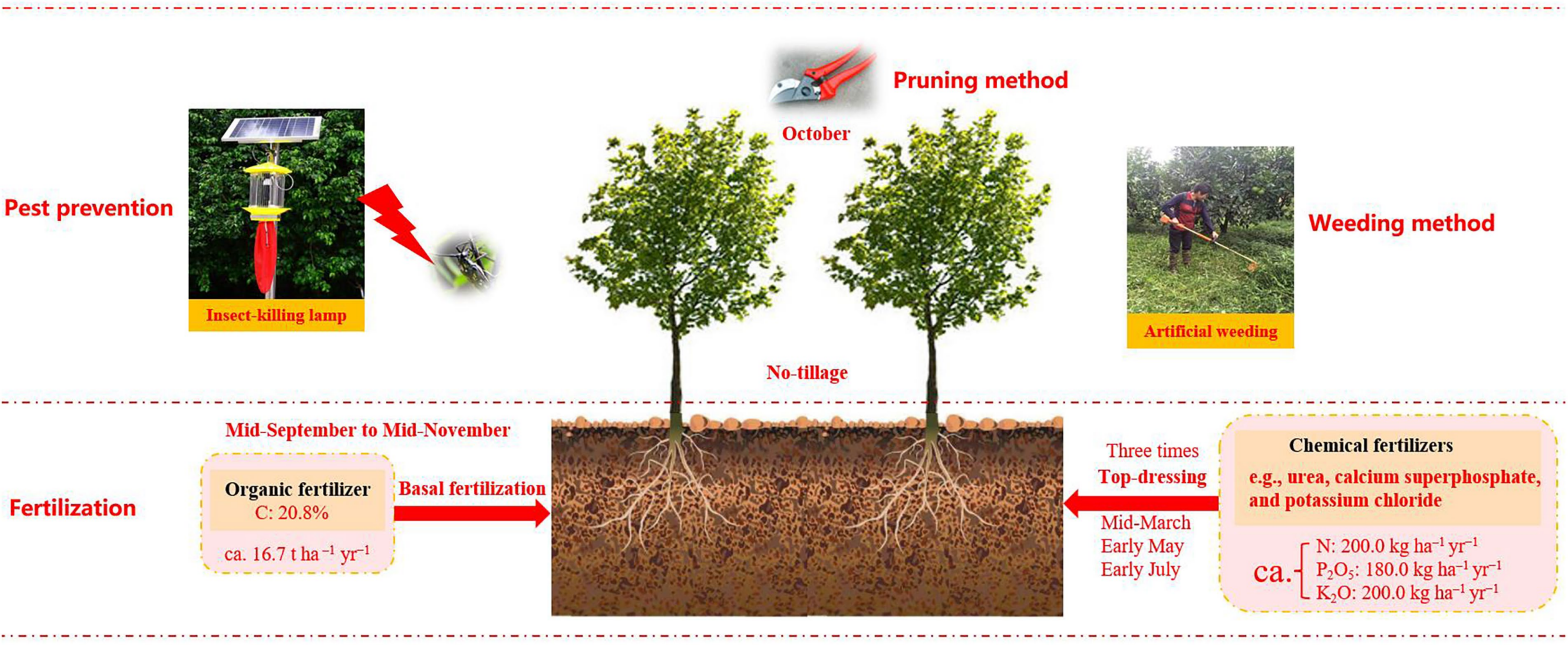
The agricultural management practices (e.g., fertilization, pest prevention and weeding method, etc.) in the present study (Luan et al., 2022).

Orchard plantations are considered a valuable resolution to land restoration by augmenting soil carbon capture and SOC stocks (Zheng et al., 2021; Luan et al., 2022). Walnut (*Juglans regia* L.), a major economic crop in many countries, has been widely grown across China, Chile, Iran, and Ukraine (Shigaeva and Darr, 2020). In 2017, global walnut production was *ca.* 3.83 million t, with China contributing a share of 50%, being the largest walnut-producing country (Meisen et al., 2020). In several poverty-stricken hilly regions in China, farmers have transformed abandoned lands into walnut plantations over the last few decades, because of the higher walnut economic values (Shigaeva and Darr, 2020), which caused there are many walnut orchards of various ages. Different chronosequence phases could affect soil microbes, EEs activities, and SOC sequestration through the walnut growth characteristics and agricultural management measures (Figure 1; Wang et al., 2017; Luan et al., 2022); however, the underlying connections among these indices remain uncertain and elusive, which might be the main impediment to SOC stocks in agroecosystems. Thus, by using the space-for-time substitution method (Sparling et al., 2003), clarifying the mechanisms of microbial-driven changes in SOC quantity and quality across different orchard ages could deeply improve our understanding of agricultural SOC sequestration and cycling. Besides, because walnuts own deep root systems (Jerszurki et al., 2017), it is imperative to illustrate the microbial mechanism for driving SOC dynamics in the soil profile.

Here, four walnut orchards of various ages [0- (i.e., wasteland), 7-, 14-, and 21-years] in the East Taihang Mountains in Hebei province were chosen to evaluate the temporal variations in the microbial communities, EEs activities, and SOC pools in the soil profile (0–20, 20–40, and 40–60 cm) and to explicit the connections among these factors. The specific aims were to (i) evaluate the impacts of walnut plantation ages on microbial communities, EEs activities, SOC quantity, and quality (as indicated by the values of lability index; see Section 2.7.2); (ii) quantify the distributions of the above-mentioned indices in different soil depths (0–20, 20–40, and 40–60 cm); and (iii) demonstrate the mechanisms of microbial-driven changes in SOC quantity and quality in the hilly region of Hebei province, China.

## Materials and methods

### Site description

This study was performed in the ‘Lijiahan’ walnut demonstration base (*ca.* 1,000 ha; longitude 114°30′–114°33′E, latitude 37°29′–37°32′N) located in Lincheng County, Hebei Province, China. This region has a warm temperate monsoon climate, with mean annual temperature and precipitation of 13.0°C and 521 mm, respectively. In addition, soils in this region are Luvisols according to the FAO Soil Taxonomy (FAO, 2014).

Considering the economic benefits of walnut, large amounts of abandoned lands have been exploited for walnut gardens since the 1960s. Therefore, walnut orchards of varying ages have widely existed in this region. The cultivation density of walnut was about 667 plants per hectare, with rows 3.0 m apart and plants 5.0 m apart. The detailed information about agricultural management measures in the study sites was shown in Figure 1.

### Experimental design

Soil variables over the walnut plantation chronosequence with similar field management measures and soil conditions were evaluated through the method of space-for-time substitution, which is a valuable way to monitor soil temporal changes (Sparling et al., 2003). The walnut plantations of varying ages in the study sites provide an ideal chance to explore soil temporal changes during the walnut cultivation process.

In this study, soil samples were collected from four walnut (variety ‘Lvling’) plantations with different ages (0-, 7-, 14-, and 21-years) in September 2020. A completely random design with three replicates was laid out for each walnut plantation (12 plots; area 225 m^2^). Four walnut plantations were situated in the same geomorphologic units (hills) with similar soil classification, slope direction and gradient, and field management measures.

### Soil sampling

In each plot, five soil cores (5 cm diameter, 20 cm depth) were randomly obtained from three soil depths (0–20, 20–40, and 40–60 cm; major distribution area of walnut root system) under walnut canopies, and then, these soil samples were integrated into one combined sample. In total, 36 combined samples (12 plots × 3 depths) were obtained. After the removal of plant residues, macrofauna, and stones, these combined soil samples were carefully passed through a sieve (2 mm) and then divided into two parts. One part was air-dried to determine soil physicochemical properties; the other part was conserved in a refrigerator (−80°C) for the measurement of soil microbial-associated characteristics.

### Soil physicochemical and microbial analysis

SOC and total N (TN) were quantified using the heated dichromate/titration method (Mebius, 1960) and the Semimicro Kjeldahl method (Bremner, 1960), respectively. Soil bulk density (BD) was assessed by the core sampling method (Andreev et al., 2016).

SOC pools were determined by the modified Walkley-Black method (Chan et al., 2001). Briefly, 5, 10, and 20 ml concentrated sulfuric acid (H2SO4) were applied to form three aqueous acid mediums with the ratios of 0.5:1, 1:1, and 2:1, which caused gradient oxidizing conditions. Based on SOC oxidizable properties, SOC is separated into four C pools with different oxidizability/lability: pool I (very labile C, CVL, C oxidized under 5 ml H2SO4), pool II (labile C, CL, the difference in C oxidized under 5 ml and 10 ml H2SO4), pool III (less labile C, CLL, the difference in C oxidized under 10 ml and 20 ml H2SO4), and pool IV (non-labile C, CNL, residual C after oxidization with 20 ml H2SO4 when compared with SOC). Among these C pools, the sum of CVL and CL constitute active C pools, while CLL and CNL are regarded as passive C pools (Liu et al., 2018).

The Phospholipid fatty acids were measured by the method described by Cui et al. (2021a) using a Gas Chromatograph (N6890, Agilent Technologies, Santa Clara, CA, United States) fitted with a MIDI Sherlock microbial identification software (Version 4.5; MIDI Inc., Newark, DE, United States). The methyl nonadecanoate (19:0) was added as an internal standard, and the following Phospholipid fatty acids (PLFA) biomarkers were chosen for assessing the soil microbial community: Gram-positive bacteria (G+; i15:0, a15:0, i16:0, a17:0 and i17:0), Gram-negative bacteria (G−; 16:1ω7c, cy17:0, 18:1ω7c, and cy19:0), arbuscular mycorrhizal fungi (AMF; 16:1ω5c), saprophytic fungi (SF; 18,2ω6c and 18:1ω9c), and actinomycetes (10Me-16,0, 10Me-17:0, and 10Me-18:0; Peng et al., 2016). The sum of G+ and G− constitutes bacteria, while the sum of AMF and SF constitutes fungi. Besides, the sum of bacteria, fungi, and actinomycetes constitutes total microorganisms. The contents of PLFAs were expressed in units of nmol g^−1^.

Soil extracellular enzyme activities were measured by the micro-plate enzyme technique (DeForest, 2009). Five labelled fluorogenic substrates based on 4-methylumbelliferyl (4-MUB) were used to determine the hydrolase activities: 4-MUB-α-D-glucoside for α-glucosidase (αG), 4-MUB-β-D-glucoside for β-glucosidase (βG), 4-MUB-β-D-cellobioside for β-cellobiosidase (CBH), 4-MUB-β-D-xyloside for β-xylosidase (βX), and 4-MUB-N-acetyl-β-D-glucosaminide for N-Acetyl-glucosaminidase (NAG). In addition, L-3, 4-dihydroxyphenylalanine substrate was used to measure the oxidase activities (phenol oxidase (PHOs) and peroxidase (PerX)). The detailed determination procedure for these enzyme activities was referred to Cui et al. (2021b). The hydrolase activities were quantified using a microplate fluorometer (Scientific Fluoroskan Ascent FL, Thermo, United States) with 365 nm excitation and 450 nm emission; meanwhile, the oxidase activities were determined using a microplate fluorometer with 450 nm absorbance. These enzyme activities were calculated and expressed as nmol g^−1^ h^−1^.

### Data calculations

#### The calculation of SOC stocks

The SOC stocks (SOCstock; Mg ha^−1^) at the three depths (0–20, 20–40, and 40–60 cm) were calculated by the following equation (Li et al., 2018):


(1) SOCstock=SOC×BD×H/10

Where BD and H represent the soil bulk density (g cm^−3^) and the depths of three soil layers (cm), respectively.

#### The calculation of SOC lability/quality

The lability index (LI), which could be applied for assessing SOC quality, was calculated as follows (Liu et al., 2018):


(2) $$\mathrm{LI}=\left(\mathrm{CVL}\times 3+\mathrm{CL}\times 2+\mathrm{CLL}\right)/\mathrm{SOC}$$

Where C_VL_, C_L_, and C_LL_ represent very labile C, labile C, and less labile C, respectively.

#### The geometric mean of the extracellular enzyme activities

The geometric mean of the assayed extracellular enzyme activities (GMEA), hydrolase (GH) and oxidase (GOR) were calculated by the following equations (Luan et al., 2022):


(3) $$\mathrm{GMEA}=\sqrt[{7}]{\alpha G\times \beta G\times \mathrm{CBH}\times \beta X\times \mathrm{NAG}\times \mathrm{PHOs}\times \mathrm{PerX}}$$

(4) $$\mathrm{GH}=\sqrt[{5}]{\alpha G\times \beta G\times \mathrm{CBH}\times \beta X\times \mathrm{NAG}}$$

(5) $$\mathrm{GOR}=\sqrt[{2}]{\mathrm{PHOs}\times \mathrm{PerX}}$$

Where αG, βG, CBH, βX, NAG, PHOs and PerX represent α-Glucosidase, β-Glucosidase, β-Cellobiosidase, β-Xylosidase, N-Acetyl-glucosaminidase, phenol oxidase, and peroxidase, respectively.

### Statistical analysis

One-way ANOVA was applied for evaluating the effect of different walnut plantation ages (or soil depths) on soil characteristics, e.g., SOC, CVL, LI, microbial properties, etc. Duncan tests were used for determining the significance level (*p* < 0.05). Two-way ANOVA was conducted for comparing the differences in these soil variables with four walnut plantation ages (0-, 7-, 14-, and 21-years) and three soil depths (0–20, 20–40, and 40–60 cm) as the main factors. SPSS 20.0 statistical software (SPSS, Chicago, United States) was used to perform the above-mentioned statistical analyses. Additionally, CANOCO software (version 5.0, Ithaca, NY) was applied for identifying the differences in microbial community across walnut orchards of contrasting ages at three soil depths through principal component analysis (PCA). The R (version 3.6.1) was used to evaluate the effects of microbial properties (e.g., fungal community, extracellular enzyme activities, etc.) on SOC quantity and quality through the partial least squares path model (PLS-PM; 1,000 bootstraps) with the packages ‘plspm’ (Sanchez et al., 2017).

## Results

### SOC contents and its stocks (SOC quantity)

The soil basic characteristics, e.g., SOC, bulk density (BD), and SOC stocks (SOCstock) at different soil depths (0–20, 20–40, and 40–60 cm), are shown in Table 1. Overall, these indices were significantly (*p* < 0.05) influenced by walnut plantation ages and soil depths. The soils in the 14-and 21-year walnut orchards exhibited higher SOC contents and SOCstock and lower BD values than those in the 0-and 7-year walnut orchards in the 0–20 and 20–40 cm soil layers, and no significant differences (*p* > 0.05) were observed in these indices among all treatments in the 40–60 cm soil layer (Table 1). Besides, irrespective of the walnut plantation ages, the SOC contents and SOCstock were reduced with increasing soil depths (0–20 cm > 20–40 cm > 40–60 cm), whereas the BD values showed an inverse trend (0–20 cm < 20–40 cm < 40–60 cm). The soils in the surface layer (0–20 cm) contained higher SOC contents and SOCstock than those in the deep layers (20–40 cm and 40–60 cm), with mean increases of 69.8 and 58.2%, respectively.

**Table 1:** Soil organic C contents and stocks at three soil depths (0–20, 20–40, and 40–60 cm) under different ages of walnut plantations.

Indices	Walnut plantation age (T; yr.)	Soil depths (D)			Effects		
		0–20 cm	20–40 cm	40–60 cm	T	D	T × D
SOC	0-year	6.09 ± 0.62 b	3.86 ± 0.32 b	3.94 ± 0.36 a	^**^	^**^	^**^
(g kg^−1^)	7-year	6.69 ± 0.74 b	4.53 ± 0.23 b	4.13 ± 0.28 a			
	14-year	7.64 ± 0.70 ab	4.69 ± 0.18 b	4.03 ± 0.38 a			
	21-year	9.23 ± 1.24 a	6.11 ± 0.87 a	3.63 ± 0.54 a			
		**7.41 A**	**4.80 B**	**3.93 B**			
BD	0-year	1.40 ± 0.03 a	1.43 ± 0.04 a	1.49 ± 0.04 a	^**^	^**^	NS
(g cm^−3^)	7-year	1.36 ± 0.04 a	1.41 ± 0.05 ab	1.46 ± 0.04 a			
	14-year	1.28 ± 0.03 b	1.36 ± 0.03 ab	1.47 ± 0.07 a			
	21-year	1.29 ± 0.05 b	1.33 ± 0.06 b	1.50 ± 0.05 a			
		**1.33 C**	**1.39 B**	**1.48 A**			
SOC_stock_	0-year	17.0 ± 1.4 b	11.1 ± 0.6 c	11.7 ± 0.8 a	^**^	^**^	^**^
(Mg ha^−1^)	7-year	18.2 ± 1.7 b	12.8 ± 0.3 b	12.1 ± 0.6 a			
	14-year	19.6 ± 2.0 ab	12.8 ± 0.3 b	11.9 ± 1.3 a			
	21-year	23.9 ± 3.7 a	16.2 ± 1.6 a	10.9 ± 1.5 a			
		**19.7 A**	**13.2 B**	**11.6 B**			

Uppercase letters in each row and lowercase letters in each column represent significant differences (*p* < 0.05) among different soil depths and different ages of walnut plantations in the same depths, respectively. ^**^ and NS represent significant at *p* < 0.01 and no significant differences (*p* > 0.05), respectively. SOC, soil organic C; BD, bulk density; and SOC_stock_, soil organic C stocks.

### SOC pools and quality

Both walnut plantation ages and soil depths strongly influenced SOC pools and quality (Table 2; Supplementary Table S1). The contents of SOC pools (i.e., CVL, CL, CLL, and CNL) were basically higher in the 14-and 21-year walnut orchards than those in the 0-and 7-year walnut orchards in the 0–20 cm (except for CNL in the 14-year walnut orchard) and 20–40 cm soil layers (except for CLL in the 14-year walnut orchard; Table 2). However, in these soil layers, we found that the 14-and 21-year walnut orchards significantly (*p* < 0.05) decreased the proportions of passive C pools, and correspondingly increased the proportions of active C pools compared to the 0-and 7-year walnut orchards (Supplementary Table S1), which caused higher values of lability index (LI) in the soils in the 14-and 21-year walnut orchards. Notably, these above-mentioned indices were observed with no significant differences (*p* > 0.05) among all treatments in the 40–60 cm soil layer and decreased with increasing soil depths (0–20 cm > 20–40 cm > 40–60 cm), except for the proportions of passive C pools that showed an inverse trend (Table 2; Supplementary Table S1).

**Table 2:** Soil organic C pools and quality/lability at three soil depths (0–20, 20–40, and 40–60 cm) under different ages of walnut plantations.

Indices	Walnut plantation age (T; yr.)	Soil depth (D)			Effects		
		0–20 cm	20–40 cm	40–60 cm	T	D	T × D
C_VL_	0-year	1.31 ± 0.05 c	0.67 ± 0.08 c	0.65 ± 0.07 a	^**^	^**^	^**^
(g kg^−1^)	7-year	1.34 ± 0.07 c	0.82 ± 0.17 bc	0.71 ± 0.07 a			
	14-year	1.81 ± 0.11 b	1.00 ± 0.09 b	0.67 ± 0.04 a			
	21-year	2.24 ± 0.21 a	1.32 ± 0.19 a	0.60 ± 0.02 a			
		**1.68 A**	**0.95 B**	**0.66 C**			
C_L_	0-year	0.84 ± 0.08 c	0.49 ± 0.04 d	0.54 ± 0.06 a	^**^	^**^	^**^
(g kg^−1^)	7-year	1.03 ± 0.21 bc	0.65 ± 0.05 c	0.55 ± 0.03 a			
	14-year	1.35 ± 0.21 ab	0.78 ± 0.03 b	0.53 ± 0.03 a			
	21-year	1.42 ± 0.22 a	0.96 ± 0.07 a	0.49 ± 0.09 a			
		**1.16 A**	**0.72 B**	**0.53 C**			
C_LL_	0-year	1.84 ± 0.06 bc	1.32 ± 0.09 c	1.30 ± 0.15 ab	^**^	^**^	^**^
(g kg^−1^)	7-year	1.80 ± 0.04 c	1.71 ± 0.08 b	1.50 ± 0.12 a			
	14-year	2.07 ± 0.14 ab	1.53 ± 0.11 bc	1.47 ± 0.17 a			
	21-year	2.30 ± 0.22 a	1.98 ± 0.22 b	1.19 ± 0.13 b			
		**2.00 A**	**1.64 B**	**1.37 B**			
C_NL_	0-year	2.09 ± 0.51 b	1.38 ± 0.14 a	1.45 ± 0.13 a	^*^	^**^	NS
(g kg^−1^)	7-year	2.51 ± 0.56 ab	1.35 ± 0.14 a	1.37 ± 0.03 a			
	14-year	2.41 ± 0.28 ab	1.38 ± 0.08 a	1.35 ± 0.16 a			
	21-year	3.26 ± 0.60 a	1.84 ± 0.50 a	1.31 ± 0.09 a			
		**2.57 A**	**1.49 B**	**1.37 B**			
LI	0-year	1.22 ± 0.07 b	0.99 ± 0.06 b	0.96 ± 0.04 a	^**^	^**^	^**^
	7-year	1.16 ± 0.06 b	1.04 ± 0.13 b	0.99 ± 0.05 a			
	14-year	1.36 ± 0.05 a	1.23 ± 0.05 a	0.97 ± 0.05 a			
	21-year	1.37 ± 0.05 a	1.24 ± 0.01 a	0.98 ± 0.04 a			
		**1.28 A**	**1.13 B**	**0.97 C**			

Uppercase letters in each row and lowercase letters in each column represent significant differences (*p* < 0.05) among different soil depths and different ages of walnut plantations in the same depths, respectively. ^*^, ^**^ and NS represent significant at *p* < 0.05, *p* < 0.01 and no significant differences (*p* > 0.05), respectively. C_VL_, very labile C; C_L_, labile C; C_LL_, less labile C; C_NL_, non-labile C; and LI, lability index.

### Soil phospholipid fatty acid profiles

**Figure 2:**
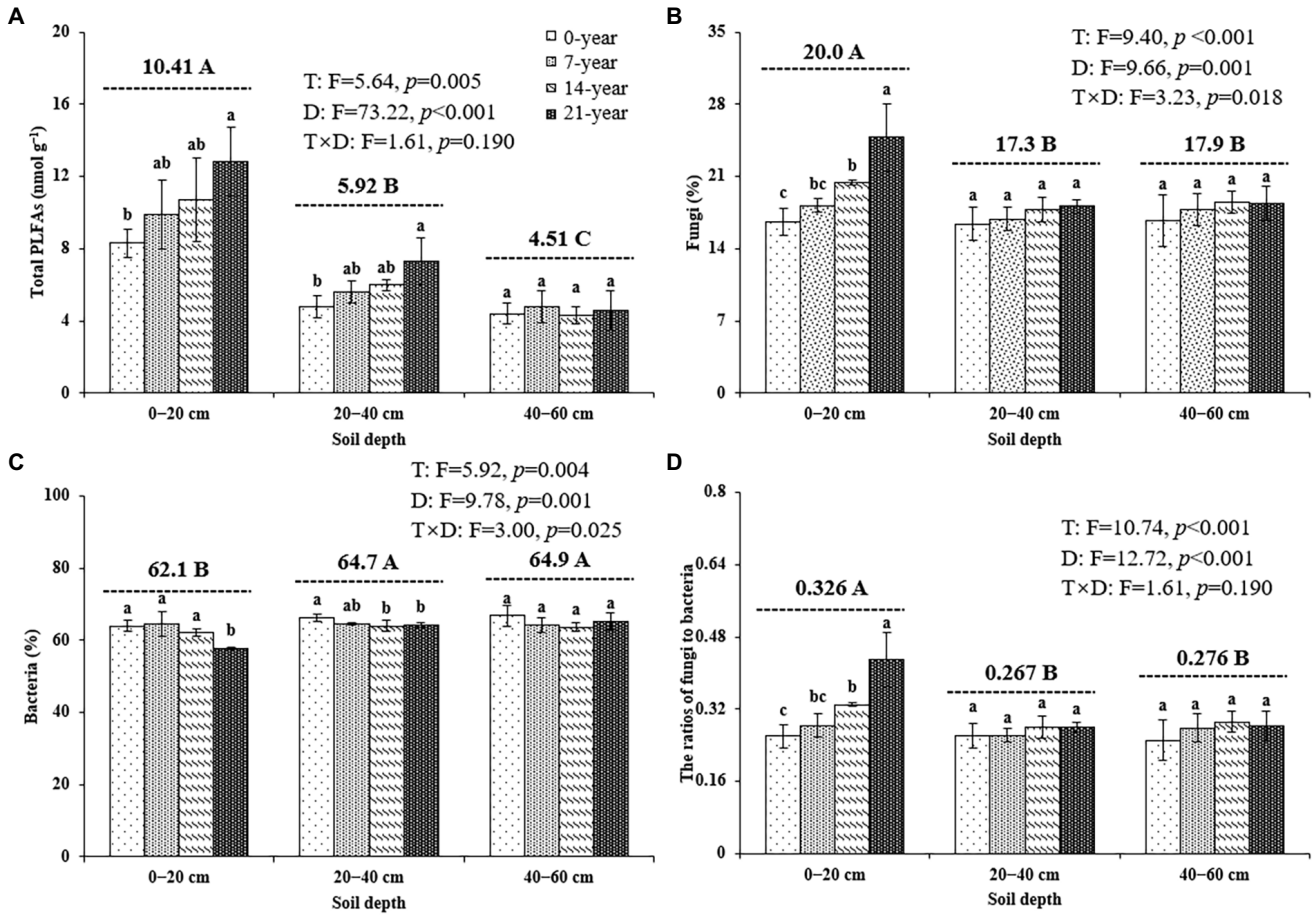
The contents of total PLFAs **(A)**, the proportions of fungi **(B)**, and bacteria **(C)** as well as the ratios of fungi to bacteria **(D)** at three soil depths (0–20, 20–40, and 40–60 cm) under different ages of walnut plantations. Uppercase and lowercase letters represent significant differences (*p* < 0.05) among different soil depths and different ages of walnut plantations in the same depths, respectively. Two-way ANOVA was applied for identifying the effects of walnut plantation ages, soil depths, and their interaction on the above indices (The data is shown in the upper right corner). T, walnut plantation ages; D, soil depths.

**Figure 3:**
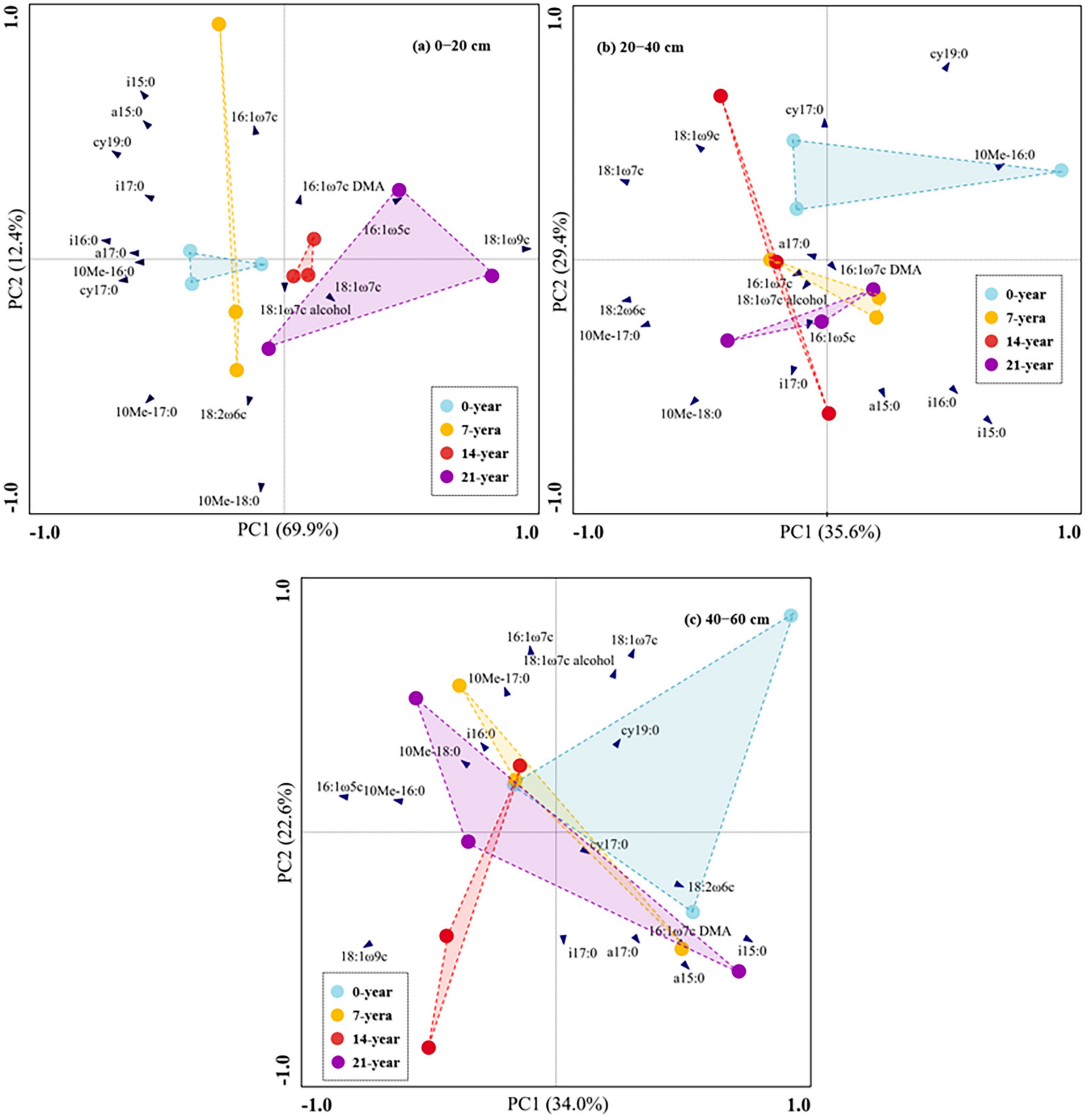
Principal component analysis (PCA) based on 19 individual PLFAs in this study across walnut orchards of contrasting ages at the three soil depths (**(A)** 0–20 cm, **(B)** 20–40 cm, and **(C)** 40–60 cm).

The soil microbial biomass (as indicated by total PLFAs) generally decreased with the soil depths (Figure 2). Also, the soils in the 14-and 21-year walnut orchards contained 7.7–54.4% and 6.8–51.9% higher microbial biomass than those in the 0-and 7-year walnut orchards in the 0–20 cm and 20–40 cm soil layers, respectively (Figure 2A). The microbial community in this study was dominated by bacteria (57.8–66.8%), followed by fungi (16.4–24.8%) and actinomycetes (16.4–19.4%) at the three soil depths (Figures 2B,C; Supplementary Table S2). The walnut plantations induced an increase in the proportions of fungi and a decrease in the proportions of bacteria (except for 7-year walnut orchard in the 0–20 cm soil layers) in soils, which resulted in higher ratios of fungi to bacteria (F/B) in the walnut plantations (Figure 2D). Notably, the soils in the surface layer showed significantly (*p* < 0.05) higher proportions of fungi (20.0%) and F/B values (0.326) and lower proportions of bacteria (62.1%) than those in the deep layers, irrespective of the walnut planting age (Figure 2). In addition, the results of PCA (Figure 3) further demonstrated that walnut plantation ages changed the profiles of microbial communities at different soil layers. The principal components (PC1 and PC2) together accounted for 82.3, 65.0, and 56.6% of the total variation of microbial community composition at three soil depths (0–20, 20–40, and 40–60 cm), respectively. The PLFA profiles in the walnut orchards had an obvious boundary with wasteland in the 0–20 cm (except for 7-year walnut orchard) and 20–40 cm soil layers, but not in the 40–60 cm soil layers, which indicated that the effects of walnut plantation ages on microbial communities were mainly concentrated in the 0–40 cm soil layers, rather than in the 40–60 cm soil layers.

### Soil extracellular enzyme activities

**Figure 4:**
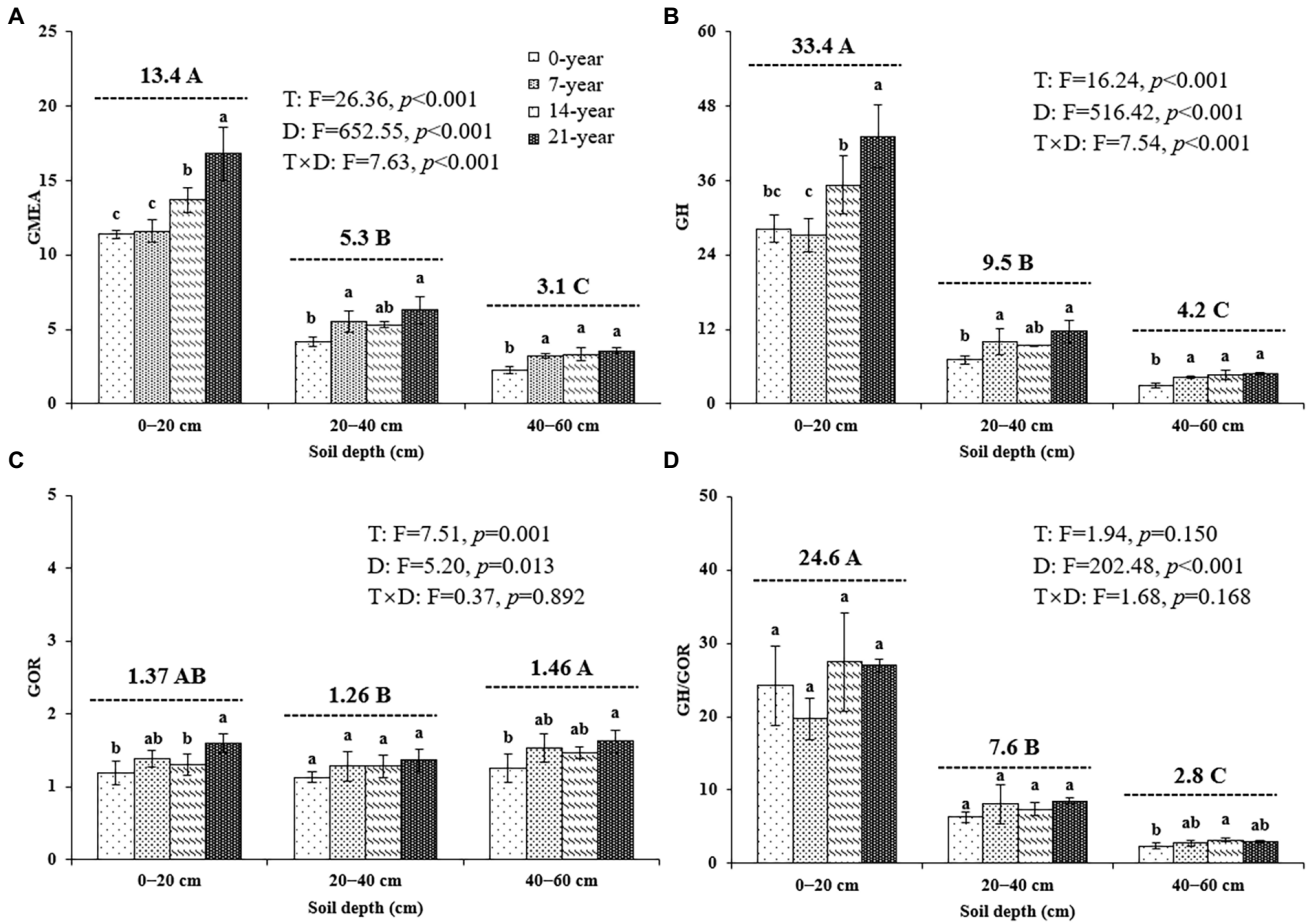
The geometric mean of the assayed extracellular enzyme activities (**(A)** GMEA, **(B)** GH, **(C)** GOR, and **(D)** GH/GOR) at three soil depths (0–20, 20–40, and 40–60 cm) under different ages of walnut plantations. Uppercase and lowercase letters represent significant differences (*p* < 0.05) among different soil depths and different ages of walnut plantations in the same depths, respectively. Two-way ANOVA was applied for identifying the effects of walnut plantation ages, soil depths, and their interaction on the above indices (The data is shown in the upper right corner). GMEA, the geometric mean of seven extracellular enzyme activities; GH, the geometric mean of the hydrolase activities; GOR, the geometric mean of the oxidase activities.

The continuous planting of walnut caused a remarkable change in soil microbial hydrolase and oxidase activities; however, the changing trends of these enzyme activities were inconsistent among different soil depths (Table 3; Figure 4). Similar trends were observed for all hydrolase activities, including αG, βG, CBH, βX, and NAG, in the surface (14-and 21-year >0-and 7-year) and deep (7-, 14-, and 21-year >0-year) soils (Table 3). Also, the oxidase activities (i.e., PHOs and PerX) in the walnut orchards were higher than those in the wasteland at different soil depths (Table 3). Notably, irrespective of the walnut plantation ages, the hydrolase activities (as indicated by the values of GH) in the surface soils seemed to be remarkably higher than those in the deep soils (average of 387.7% increase); meanwhile, the oxidase activities (as indicated by the values of GOR) in the 40–60 cm soil layers were significantly (*p* < 0.05) higher by 7.1–14.0% than those in other soil layers (Figure 4).

**Table 3:** The extracellular enzyme activities (g^−1^ soil h^−1^) at three soil depths (0–20, 20–40, and 40–60 cm) under different ages of walnut plantations.

Indices	Walnut plantation age (T; yr.)	Soil depth (D)			Effects		
		0–20 cm	20–40 cm	40–60 cm	T	D	T × D
αG	0-year	17.1 ± 2.6 b	4.4 ± 0.7 a	2.3 ± 0.5 b	^**^	^**^	^**^
	7-year	20.1 ± 2.1 b	6.1 ± 1.8 a	3.7 ± 0.6 a			
	14-year	22.1 ± 1.4 b	5.5 ± 0.4 a	3.3 ± 0.9 ab			
	21-year	32.1 ± 5.3 a	6.8 ± 1.7 a	4.4 ± 0.7 a			
		**22.8 A**	**5.7 B**	**3.4 B**			
βG	0-year	136.7 ± 7.1 b	27.8 ± 3.8 b	22.3 ± 2.7 a	^**^	^**^	^**^
	7-year	124.2 ± 15.3 b	37.0 ± 7.4 ab	25.5 ± 6.3 a			
	14-year	161.7 ± 20.5 ab	34.3 ± 5.5 ab	26.6 ± 6.0 a			
	21-year	191.6 ± 43.5 a	43.4 ± 7.1 a	30.5 ± 4.4 a			
		**153.6 A**	**35.6 B**	**26.2 B**			
CBH	0-year	19.8 ± 4.5 b	2.9 ± 0.9 a	0.9 ± 0.4 b	^**^	^**^	^**^
	7-year	20.6 ± 3.6 b	4.0 ± 1.5 a	1.5 ± 0.2 a			
	14-year	26.5 ± 7.4 b	3.8 ± 0.5 a	1.8 ± 0.1 a			
	21-year	37.1 ± 5.0 a	4.0 ± 2.0 a	1.7 ± 0.3 a			
		**26.0 A**	**3.7 B**	**1.5 B**			
βX	0-year	40.6 ± 7.2 ab	13.3 ± 1.0 b	2.6 ± 0.4 c	^**^	^**^	^**^
	7-year	30.3 ± 5.6 b	25.7 ± 5.7 a	3.9 ± 0.3 b			
	14-year	41.8 ± 7.4 ab	21.8 ± 4.5 a	4.8 ± 0.9 ab			
	21-year	44.0 ± 4.6 a	29.2 ± 4.7 a	5.3 ± 0.3 a			
		**39.2 A**	**22.5 B**	**4.1 C**			
NAG	0-year	10.0 ± 2.5 b	3.9 ± 0.5 b	1.8 ± 0.6 a	^**^	^**^	^**^
	7-year	9.6 ± 0.9 b	4.5 ± 0.8 b	2.8 ± 0.5 a			
	14-year	13.9 ± 0.6 a	4.8 ± 0.4 b	2.9 ± 0.7 a			
	21-year	15.1 ± 2.7 a	6.9 ± 1.0 a	2.5 ± 0.5 a			
		**12.1 A**	**5.0 B**	**2.5 C**			
PHOs	0-year	1.22 ± 0.14 b	1.13 ± 0.14 a	1.33 ± 0.20 a	NS	^*^	NS
	7-year	1.39 ± 0.18 ab	1.30 ± 0.22 a	1.48 ± 0.29 a			
	14-year	1.41 ± 0.15 ab	1.26 ± 0.12 a	1.46 ± 0.17 a			
	21-year	1.60 ± 0.07 a	1.28 ± 0.18 a	1.45 ± 0.20 a			
		**1.40 A**	**1.24 B**	**1.43 A**			
PerX	0-year	1.16 ± 0.18 b	1.13 ± 0.14 a	1.18 ± 0.28 b	^**^	^*^	NS
	7-year	1.39 ± 0.17 ab	1.26 ± 0.20 a	1.61 ± 0.21 ab			
	14-year	1.21 ± 0.15 b	1.31 ± 0.21 a	1.47 ± 0.28 ab			
	21-year	1.59 ± 0.20 a	1.45 ± 0.20 a	1.82 ± 0.13 a			
		**1.34 AB**	**1.29 B**	**1.52 A**			

Uppercase letters in each row and lowercase letters in each column represent significant differences (*p* < 0.05) among different soil depths and different ages of walnut plantations in the same depths, respectively. ^**^, ^*^ and NS represent significant at *p* < 0.01, *p* < 0.05 and no significant differences (*p* > 0.05), respectively. αG, α-Glucosidase; βG, β-Glucosidase; CBH, β-Cellobiosidase; βX, β-Xylosidase; NAG, N-Acetyl-glucosaminidase; PHOs, phenol oxidase; and PerX, peroxidase.

### Correlations among microbial characteristics, SOC quantity, and quality

**Figure 5:**
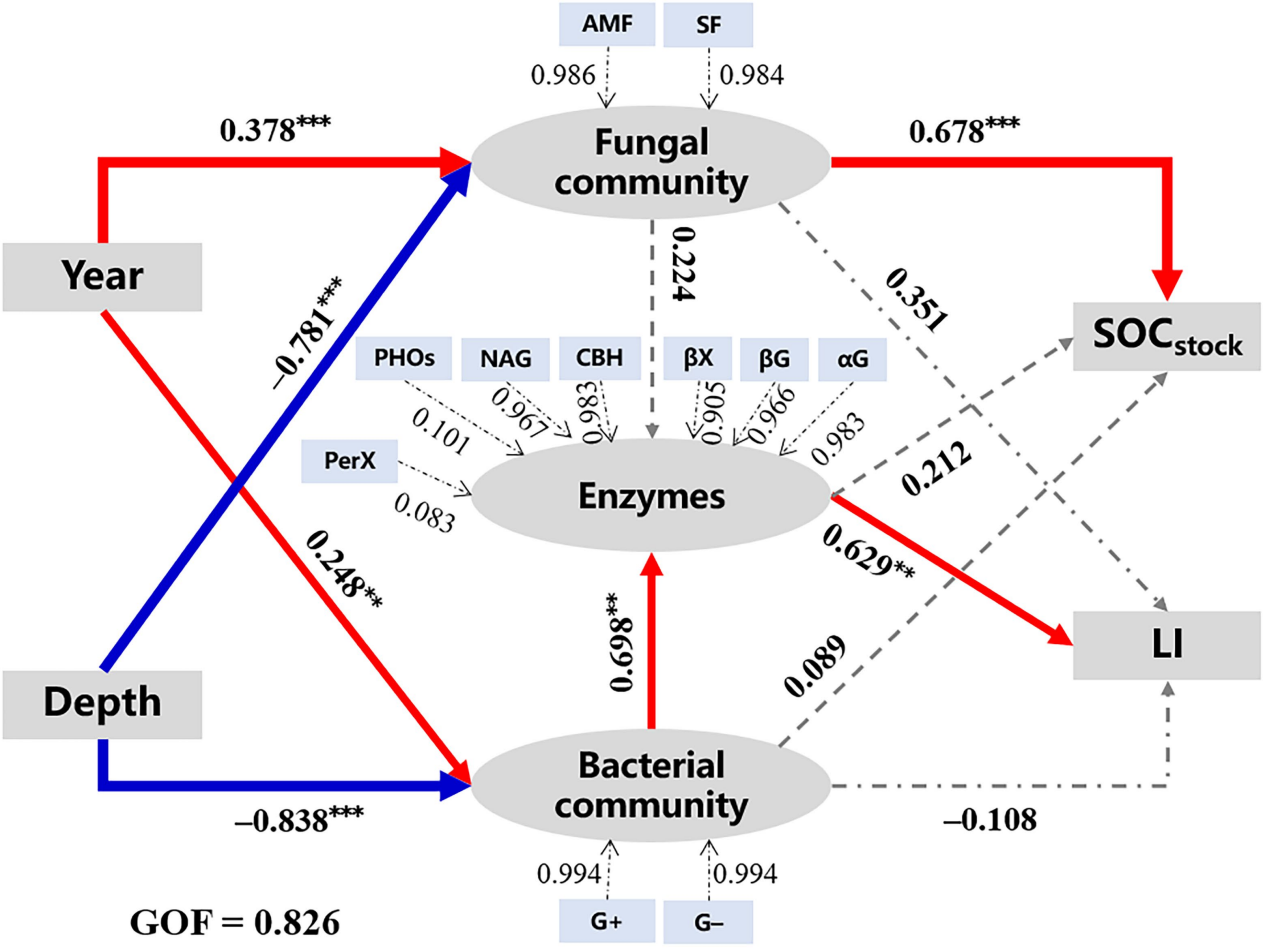
The PLS-PM analysis demonstrates how walnut plantation ages and soil depths affected the quantity and quality of SOC by regulating microbial characteristics. The red lines represent positive effects, while the blue lines represent negative effects. Besides, the grey lines represent non-significant effects (*p* > 0.05). The widths of the lines represent the strength of the effects. The values above the arrows represent path coefficients (^**^p < 0.01 and ^***^p < 0.001). The values of goodness of fit (GOF) denote the goodness of fit index. G+, Gram-positive bacteria; G−, Gram-negative bacteria; AMF, arbuscular mycorrhizal fungi; SF, saprotrophic fungi: other abbreviations see Tables 1–3.

The PLS-PM analysis (GOF = 0.826) was applied to explore the correlations among microbial characteristics, SOC quantity, and quality (Figure 5). Results showed that walnut plantation ages (path coefficient = 0.378^***^ and 0.248^**^) and soil depths (path coefficient = −0.781^***^ and − 0.838^***^) had strongly positive and negative effects on microbial communities (i.e., fungal and bacterial communities), respectively, which were consistent with the results of Figures 2, 3. Moreover, bacterial community (0.698^***^), rather than fungal community (0.224; *p* > 0.05), was regarded as an important factor that regulated enzyme activities. Subsequently, the fungal community (0.678^***^) had positively direct effects on SOC stocks; meanwhile, the bacterial community indirectly affected SOC quality/liability (as indicated by the values of LI) through its direct influences on enzyme activities.

## Discussion

### Variations in soil microbial communities

Microbes are one of the vital biological parts of soils (Song et al., 2020); meanwhile, they can provide more important information involved in variations in soil microenvironment than physicochemical characteristics (Blagodatskaya and Kuzyakov, 2013; Escalas et al., 2019). Our findings demonstrated that successive walnut planting was beneficial for microbial growth (as indicated by the contents of total PLFAs) in the 0–40 cm soil layers (Figure 2), which were confirmed by the findings of the other studies that long-term Chinese fir plantations (26-year) and citrus orchard plantations (40-year) also promoted microbial growth in Guangxi and Jiangxi Province, China, respectively (Mao et al., 2021; Zheng et al., 2022). The likely reasons were that the orchard soils contained more ‘food’ (e.g., C and nutrients; Table 1; Supplementary Table S1) and showed better ‘microsites’ (e.g., high soil porosity; Supplementary Table S3) due to the continuous organic inputs (e.g., organic fertilizers and crop residues, etc.), which were more suitable for microorganisms (Wang et al., 2018; Lu et al., 2019). Besides, we found that the orchard soils were more suitable for fungal growth, rather than bacterial growth, than wastelands in the 0–40 cm soil layers (as indicated by the higher F/B ratios; Figure 2), which were explained by the microbial ‘inherent characteristics’ (Rashid et al., 2016) and the differences in soil properties among all treatments. Dai et al. (2017) revealed that fungi own a higher C/N ratio than bacteria (15 vs. 6), indicating that fungi need less N per biomass unit and are better adapted to the soils with a high SOC/TN ratio than bacteria. Zhou et al. (2016) found that soils with high porosity and low pH are more favorable to fungi than bacteria, due to the discrepancy in fungal and bacterial physiological characteristics. Therefore, the orchard soils with high SOC/TN ratios and porosity (Supplementary Table S3) could create a more suitable microenvironment for fungal growth than bacteria, which caused a higher F/B ratio in the orchard soils. The above-mentioned findings, together with our results (i.e., soil physicochemical properties were unchanged among all treatments in the 40–60 cm soil layers; Table 1; Supplementary Table S1), also explained that the microbial indices (e.g., total PLFAs and the F/B ratios; Figures 2, 3) had no obvious differences among all treatments in the 40–60 cm soil layers.

Notably, irrespective of the walnut planting age, the total microbial biomass decreased significantly with increasing soil depths in the present study (Figure 2). Several studies could support and explain this finding, indicating that the deep soils are characterized by ‘poor’ nutrient conditions and ‘densification’ (i.e., oxygen deficiency; Laskar et al., 2021; Liu et al., 2021), which were not conducive to microbial growth. Besides, we found that the F/B ratios were significantly higher in the surface soils than in the deep soils (Figure 2), consistent with higher SOC/TN ratios and total porosity in the surface soils (Supplementary Table S3). These discrepancies in SOC/TN ratios and total porosity could also explain the changes in F/B ratios among different soil layers (Zhou et al., 2016; Dai et al., 2017).

### Variations in soil extracellular enzyme activities

The EEs involved in soil C cycling, vital indicators of soil microbial activity (Luo et al., 2017), were sensitive to agricultural management practices (e.g., fertilization and tillage, etc.; He et al., 2021; Cui et al., 2021b). As previously observed (Wang et al., 2021; Zheng et al., 2022), the activities of αG and βG generally showed increasing tendencies in older Cunninghamia and citrus plantations, respectively, which was similar to the results (Table 3; Figure 4) in the present study. The possible explanation for these findings is that the higher microbial biomass in the orchard soils (Figure 2) could be beneficial for augmenting EEs activities through microbial secretion and production (Jastrow et al., 2007; Luo et al., 2017); meanwhile, the plentiful C resources (EEs-substrates; Table 1) in the orchard soils also partially explained these findings (Cenini et al., 2016).

As shown in Table 3 and Figure 4, the hydrolase activities were significantly decreased with increasing soil depths. This was supported and explained by the findings of Stone et al. (2014) and Lyu et al. (2021), who indicated that the decrease in these indices with soil depths is primarily related to changes in SOC quality, mainly in the amount of labile C resources. Namely, the higher SOC lability (i.e., higher proportions of labile C; see Table 2) in the surface soils is favorable for microbial hydrolytic function (Burns et al., 2013). In contrast, the oxidase activities were higher in the 40–60 cm soil layer than in other soil layers (Table 3; Figure 4), which were consistent with the results obtained by Sinsabaugh (2010) and Mazzon et al. (2018), who suggested that the deep soil layers owned higher oxidase activities than the surface soil layers. These findings could be explained by the differences in the edaphic factors, e.g., pH, soil compactness, SOC characteristics, as well as in enzyme’s functions (Piotrowska-Długosz et al., 2022). Zeglin et al. (2007) and Burns et al. (2013) revealed that oxidases mainly participated in stable C fractions decomposition and their activities could be inhibited by the higher N levels in soils. Thus, the higher proportions of stable C (Supplementary Table S1) and lower N levels (Supplementary Table S3) in the deep soils (40–60 cm) are conducive to microbial oxidative function.

### Variations in SOC quantity (stocks) and quality (lability)

SOC storage is a dynamic equilibrium of C inputs (e.g., organic inputs) and outputs (e.g., mineralization), and it is altered through a series of microbial processes (e.g., synthesis and decomposition; Li et al., 2020). Our results suggested that successive walnut plantations could hold a more labile/active C pool and a larger reserve of stable/passive C pool (Table 2), which is conducive to both short-term C cycle to improve agricultural productivity and long-term C storage to alleviate climate warming (Amelung et al., 2020). The higher SOC stocks in the orchard soils (0–40 cm soil layers; Table 1) could be partially ascribed to the addition of C resources input (e.g., organic fertilizers and crop residues) in the present study (Liu et al., 2018; Luan et al., 2022). Besides, Smith et al. (2014) reported that a fungal-dominated microbial community was beneficial for SOC stocks, due to fungal physiological characteristics (e.g., higher C utilization/assimilation efficiency and more stable C metabolites; Kallenbach et al., 2015). This finding was supported by the PLS-PM results (Figure 5), i.e., the fungal community had positive effects on SOC stocks. Notably, a key finding of our study is that long-term walnut plantations are helpful to not only increase SOC stocks, but also improve SOC quality (i.e., increase SOC lability) in the 0–40 cm soil layers (Table 2). Higher organic inputs (e.g., organic fertilizers and crop residues; contain more labile C resources; Elzobair et al., 2016) in the orchard soils has caused SOC dominated by ‘fresh’ and labile C resources, hereby increasing SOC lability to some extent. Wang et al. (2016) and Zheng et al. (2021) found that the conversion of wasteland to orchard can alter the quantity and quality of C resource inputs, and subsequently affect SOC composition and lability through soil microbial community. Our results (Figure 5) confirmed these findings and revealed that bacterial community, rather than fungal community, could influence SOC quality/lability (as indicated by the values of LI) through microbial functions (i.e., enzymes; Guo et al., 2018; Duan et al., 2021).

In accordance with previous studies (Hu et al., 2021; Lyu et al., 2021), we also found that with an increase in soil depths, the SOC stocks and their lability/quality decreased gradually (Tables 1, 2). The higher SOC stocks in the surface soils could be related to the microbial characteristics and the location of organic inputs (e.g., organic fertilizers and crop residues) in the present study. As shown in section 4.1, the surface soils are ‘microbial hotspots’ (Figures 2, 4; Xu et al., 2013), as exogenous C resources can be utilized quickly by microbes before these C resources reach deeper soils (Jiao et al., 2020; Ni et al., 2020). The deep soil physical characteristics (high densification and compactness; i.e., high BD values; Table 1) can prevent exogenous C into the deeper soil layers (Laskar et al., 2021), thus resulting in lower SOC stocks in the deep soils (Table 1). Besides, the C inputs to surface soils are mainly organic fertilizers, crop residues, and root exudates (i.e., labile C resources), while the C inputs to deep soils are primarily decomposed and microbially derived C (i.e., stable C resources; Elzobair et al., 2016; Ni et al., 2020), which could be the reason for the changes in SOC lability among different soil depths (Table 2). The lower SOC/TN ratios (an index for evaluating SOC degradation degree; Kiem and Kögel-Knabner, 2002) in deep soils also confirmed these SOC are characterized as ‘high degradation degree’.

## Conclusion

This study integrated several methods (i.e., the modified Walkley-Black method, PLFAs analysis, and micro-plate enzyme technique) to evaluate the spatial–temporal variations of SOC-and microbial-related indices, and gain insights into the mechanisms of microbial-mediated SOC quantity and quality in the process of walnut plantations in China. Firstly, we confirmed that long-term walnut plantations were beneficial for enlarging SOC stocks, improving SOC quality/lability, and promoting microbial growth (e.g., fungal and bacterial growth) and functions (i.e., hydrolytic and oxidative functions); meanwhile, the changes in these SOC-and microbial-related indices among walnut orchards of varying ages were mainly occurred in the 0–40 cm soil layers, rather than in 40–60 cm soil layers. Besides, these indices (e.g., total PLFAs and microbial hydrolytic function, etc.) were reduced with increasing soil depths (0–20 cm > 20–40 cm > 40–60 cm), while microbial oxidative function showed a different trend (0–40 cm < 40–60 cm). More importantly, the PLS-PM model revealed that the fungal community is the critical factor that regulates SOC stocks; meanwhile, the bacterial community affects SOC quality/lability through their controls on enzyme activities. This study’s findings can offer several valuable insights into microbial-mediated SOC dynamics in the walnut garden ecosystems in China.

## Data availability statement

The original contributions presented in the study are included in the article/Supplementary material, further inquiries can be directed to the corresponding authors.

## Author contributions

SG, XuZ, and GQ conceived and designed the study. HL, YL, SH, and WQ collected soil samples. HL, YL, SH, TG, and WQ performed soil indices analyses. HL, JC and YL analyzed and interpreted the data with support from SH, WQ, and XiZ. HL and YL wrote the manuscript with input from other authors. All authors contributed to the article and approved the submitted version.

## Funding

This study was financially supported by the Natural Science Foundation of Hebei Province (C2021204160), HAAFS Science and Technology Innovation Special Project (2022KJCXZX-ZHS-6), the Scientific research project of colleges and universities in Hebei Province (QN2022022), the Scientific Research Projects for Talents Introduce in Hebei Agricultural University (YJ2020054 and YJ2020058), and the Science and technology planning project in Hebei province (21326304D-2).

## Conflict of interest

The authors declare that the research was conducted in the absence of any commercial or financial relationships that could be construed as a potential conflict of interest.

## Publisher’s note

All claims expressed in this article are solely those of the authors and do not necessarily represent those of their affiliated organizations, or those of the publisher, the editors and the reviewers. Any product that may be evaluated in this article, or claim that may be made by its manufacturer, is not guaranteed or endorsed by the publisher.
