# Supplementary material for: Successive walnut plantations alter soil carbon quantity and quality by modifying microbial communities and enzyme activities
Source: Front Microbiol. 2022 Jul 25;13:953552. doi: 10.3389/fmicb.2022.953552 (PMC9358653; doi:10.3389/fmicb.2022.953552)
Supplement: Supplementary file 1 [file Data_Sheet_1.docx]

Table S1 The proportions of active C pools and passive C pools at three soil depths (0–20, 20–40, and 40–60 cm) under different ages of walnut plantations.

| Indices | Walnut plantation age (T; yr.) | Soil depth (D) | | |  | Effects | | |
| --- | --- | --- | --- | --- | --- | --- | --- | --- |
|  |  | 0–20 cm | 20–40 cm | 40–60 cm |  | T | D | T × D |
| Active C pools | 0-year | 35.5 ± 1.6 b | 30.1 ± 0.8 b | 30.1 ± 1.5 a |  | ** | ** | ** |
| (%) | 7-year | 35.5 ± 2.4 b | 32.4 ± 2.7 b | 30.4 ± 0.4 a |  |  |  |  |
|  | 14-year | 41.3 ± 1.6 a | 38.0 ± 1.0 a | 30.0 ± 1.4 a |  |  |  |  |
|  | 21-year | 39.8 ± 0.8 a | 37.5 ± 1.6 a | 30.3 ± 1.4 a |  |  |  |  |
|  |  | **38.0 A** | **34.5 B** | **30.2 C** |  |  |  |  |
| Passive C pools | 0-year | 64.5 ± 1.6 a | 69.9 ± 0.8 a | 69.9 ± 1.5 a |  | ** | ** | ** |
| (%) | 7-year | 64.5 ± 2.4 a | 67.6 ± 2.7 a | 69.6 ± 0.4 a |  |  |  |  |
|  | 14-year | 58.7 ± 1.6 b | 62.0 ± 1.0 b | 70.0 ± 1.4 a |  |  |  |  |
|  | 21-year | 60.2 ± 0.8 b | 62.5 ± 1.6 b | 69.7 ± 1.4 a |  |  |  |  |
|  |  | **62.0 C** | **65.5 B** | **69.8 A** |  |  |  |  |

^#^ The values are mean ± standard error (n = 3). Uppercase letters in each row and lowercase letters in each column represent significant differences (*P* < 0.05) among different soil depths and different ages of walnut plantations in the same depths, respectively. ** represents significant at *P* < 0.01.

Table S2 The proportions of actinomycetes (%) at three soil depths (0–20, 20–40, and 40–60 cm) under different ages of walnut plantations.

| Walnut plantation age (T; yr.) | Soil depth (D) | | |  | Effects | | |
| --- | --- | --- | --- | --- | --- | --- | --- |
|  | 0–20 cm | 20–40 cm | 40–60 cm |  | T | D | T × D |
| 0-year | 19.4 ± 0.7 a | 17.3 ± 0.6 a | 16.4 ± 1.5 a |  | NS | NS | ** |
| 7-year | 17.3 ± 2.8 a | 18.6 ± 1.2 a | 18.0 ± 1.0 a |  |  |  |  |
| 14-year | 17.5 ± 1.0 a | 18.2 ± 0.4 a | 18.0 ± 0.5 a |  |  |  |  |
| 21-year | 17.4 ± 2.9 a | 17.7 ± 1.0 a | 16.4 ± 2.1 a |  |  |  |  |
|  | **17.9 A** | **18.0 A** | **17.2 A** |  |  |  |  |

^#^ The values are mean ± standard error (n = 3). Uppercase letters in each row and lowercase letters in each column represent significant differences (*P* < 0.05) among different soil depths and different ages of walnut plantations in the same depths, respectively. ** and NS represent significant at *P* < 0.01 and no significant differences (*P* > 0.05), respectively.

Table S3 The other physicochemical properties at three soil depths (0–20, 20–40, and 40–60 cm) under different ages of walnut plantations.

| Indices | Walnut plantation age (T; yr.) | Soil depth (D) | | |  | Effects | | |
| --- | --- | --- | --- | --- | --- | --- | --- | --- |
|  |  | 0–20 cm | 20–40 cm | 40–60 cm |  | T | D | T × D |
| TN | 0-year | 1.15 ± 0.10 b | 0.78 ± 0.04 b | 0.80 ± 0.06 a |  | * | ** | NS |
| (g kg^−1^) | 7-year | 1.08 ± 0.05 b | 0.81 ± 0.07 b | 0.76 ± 0.06 a |  |  |  |  |
|  | 14-year | 1.26 ± 0.07 ab | 0.89 ± 0.14 ab | 0.78 ± 0.10 a |  |  |  |  |
|  | 21-year | 1.46 ± 0.17 a | 1.11 ± 0.25 a | 0.82 ± 0.10 a |  |  |  |  |
|  |  | **1.24 A** | **0.90 B** | **0.79 B** |  |  |  |  |
| Available P | 0-year | 32.6 ± 11.0 b | 17.8 ± 5.2 b | 6.7 ± 2.2 b |  | ** | ** | * |
| (mg kg^−1^) | 7-year | 29.9 ± 7.4 b | 26.3 ± 4.3 b | 14.3 ± 5.2 a |  |  |  |  |
|  | 14-year | 64.4 ± 2.9 a | 41.1 ± 6.7 a | 21.1 ± 3.8 a |  |  |  |  |
|  | 21-year | 54.4 ± 12.6 a | 36.6 ± 2.4 a | 19.3 ± 4.0 a |  |  |  |  |
|  |  | **45.3 A** | **30.4 B** | **15.3 C** |  |  |  |  |
| Available K | 0-year | 77.1 ± 2.8 b | 61.2 ± 8.1 b | 63.6 ± 4.1 a |  | ** | ** | ** |
| (mg kg^−1^) | 7-year | 120.8 ± 17.8 a | 73.6 ± 7.8 ab | 69.3 ± 3.2 a |  |  |  |  |
|  | 14-year | 135.0 ± 14.4 a | 78.9 ± 2.8 a | 68.2 ± 8.5 a |  |  |  |  |
|  | 21-year | 141.9 ± 30.5 a | 85.4 ± 13.2 a | 65.9 ± 5.6 a |  |  |  |  |
|  |  | **118.7 A** | **74.8 B** | **66.7 B** |  |  |  |  |
| SOC/TN | 0-year | 5.30 ± 0.07 b | 5.01 ± 0.71 a | 4.91 ± 0.35 a |  | NS | ** | NS |
|  | 7-year | 6.20 ± 0.41 a | 5.60 ± 0.52 a | 5.48 ± 0.20 a |  |  |  |  |
|  | 14-year | 6.06 ± 0.51 a | 5.33 ± 0.58 a | 5.21 ± 0.20 a |  |  |  |  |
|  | 21-year | 6.31 ± 0.15 a | 5.58 ± 0.46 a | 4.42 ± 0.30 a |  |  |  |  |
|  |  | **5.96 A** | **5.38 B** | **5.01 C** |  |  |  |  |
| TPo | 0-year | 47.2 ± 1.2 b | 45.9 ± 1.5 b | 43.7 ± 1.5 a |  | ** | ** | NS |
| (%) | 7-year | 48.5 ± 1.3 b | 46.8 ± 1.8 ab | 44.9 ± 1.4 a |  |  |  |  |
|  | 14-year | 51.7 ± 1.0 a | 48.5 ± 1.3 ab | 44.4 ± 2.5 a |  |  |  |  |
|  | 21-year | 51.3 ± 2.0 a | 49.7 ± 2.2 a | 43.3 ± 1.8 a |  |  |  |  |
|  |  | **49.7 A** | **47.7 B** | **44.1 C** |  |  |  |  |

^#^ The values are mean ± standard error (n = 3). Uppercase letters in each row and lowercase letters in each column represent significant differences (*P* < 0.05) among different soil depths and different ages of walnut plantations in the same depths, respectively. **, * and NS represent significant at *P* < 0.01, *P* < 0.05 and no significant differences (*P* > 0.05), respectively. SOC, soil organic C; TN, total N; TPo, total porosity.
